# GLP‐1RA use improves outcomes post partial nephrectomy in T2DM patients with RCC: A TriNetX study

**DOI:** 10.1002/bco2.70169

**Published:** 2026-02-02

**Authors:** Sam Kwon, Fiona Wardrop, Diego Gonzalez, Francis Ryan, Liza Khutsishvili, Rollins Turner, Mohammad Ghassab Deameh, Michael J. Whalen

**Affiliations:** ^1^ The George Washington University School of Medicine and Health Sciences Washington District of Columbia USA; ^2^ Faculty of Medicine Al‐Balqa Applied University As‐Salt Jordan; ^3^ Department of Urology The George Washington University School of Medicine and Health Sciences Washington District of Columbia USA

**Keywords:** diabetes mellitus, type 2, glucagon‐like peptide‐1 receptor agonists, partial nephrectomy, postoperative outcomes, renal cell carcinoma

## Abstract

**Objectives:**

The purpose of this study is to investigate the impact of Glucagon‐like peptide‐1 receptor agonists (GLP‐1RAs) use on 90‐day postoperative outcomes and overall survival following partial nephrectomy (PN) for renal cell carcinoma (RCC), where type 2 diabetes mellitus (T2DM) is a common comorbidity.

**Materials and Methods:**

The TriNetX database was used to retrospectively identify T2DM patients who underwent PN. Patients prescribed GLP‐1RAs were identified and matched in a 1:1 ratio with control patients who were not prescribed GLP‐1RAs based on baseline characteristics, comorbidities, metformin and insulin prescription, and renal function laboratory values. For 90‐day postoperative adverse events, risk difference, risk ratio and odds ratio with 95% confidence intervals were calculated. Kaplan–Meier analysis, log‐rank tests and multivariable Cox Proportional Hazards model were performed to measure the effect of GLP‐1RAs use on overall survival.

**Results:**

Use of GLP‐1RAs was associated with lower odds of acute kidney injury (odds ratio [OR] = 0.712), arrhythmia (OR = 0.725), readmission (OR = 0.8) and ileus (OR = 0.336) compared to the non‐GLP‐1RAs group (*p* < 0.05 for each). Kaplan–Meier analysis and log‐rank tests demonstrated improved 2‐year (*p* = 0.002) and 3‐year (*p* = 0.001) overall survival among patients prescribed GLP‐1RAs compared to those who were not.

**Conclusion:**

Use of GLP‐1RAs was associated with reduced incidence of 90‐day postoperative complications, such as acute kidney injury, arrhythmia, readmission and ileus, potentially contributing to improved overall survival. These results align with ongoing studies investigating the broader benefits of the use of GLP‐1RAs.

## 1 INTRODUCTION

Type 2 diabetes mellitus (T2DM) is a prevalent metabolic disease affecting over 460 million individuals worldwide.^1^ It is a common comorbidity in patients with renal cell carcinoma (RCC), with approximately 15–25% of RCC patients having pre‐existing diabetes mellitus (DM).^2^, ^3^ Among patients undergoing partial and radical nephrectomy, those with DM are associated with worse postoperative renal functional outcomes, as well as poorer cancer‐specific and overall survival.^4^, ^5^ In parallel, growing studies suggest that antidiabetic medications may impact oncologic outcomes in RCC. Studies found that metformin use has been associated with improved overall survival and cancer‐specific survival in DM patients with localised RCC^6^, ^7^ while another study suggests that dipeptidyl peptidase IV inhibitors (DPP4i) do not appear to provide clinical benefit in DM patients with metastatic RCC.^8^ These findings highlight the need for further investigation into the impact of DM medication regimens on postoperative and oncologic outcomes in RCC.

Glucagon‐like peptide‐1 receptor agonists (GLP‐1RAs) are a relatively new and evolving class of medications used to manage DM. This class includes liraglutide, exenatide, dulaglutide, semaglutide, lixisenatide and tirzepatide. By mimicking the action of endogenous glucagon‐like peptide‐1, GLP‐1RAs increase insulin secretion and suppress glucagon release.^9^ Although their mechanisms are not fully understood, early evidence suggests that GLP‐1RAs may exert renoprotective effects by activating GLP‐1 receptors on renal and immune cells, thereby reducing expression of proinflammatory and profibrotic factors.^10^

While prior studies have examined the effects of other antidiabetic medications, such as metformin and DPP4i, no studies to date have investigated the impact of GLP‐1RAs on postoperative and oncologic outcomes following renal surgery in DM patients. Therefore, the purpose of this study is to utilise the TriNetX database to evaluate the association between GLP‐1RAs use and 90‐days postoperative adverse outcomes and overall survival in T2DM patients with localised RCC undergoing partial nephrectomy (PN).

## 2 MATERIALS AND METHODS

### 2.1 Database and cohort selection

This retrospective study utilised the TriNetX Database, which is a large, global database providing access to de‐identified electronic medical records from approximately 148 million patients across 106 health‐care organisations. The study period spanned from 1 January 2005 to 15 November 2025. This period was selected given that GLP‐1RAs received Food and Drug Administration (FDA) approval in 2005.

Patients diagnosed with T2DM and localised RCC amenable to PN were identified using Current Procedural Terminology (CPT) and International Classification of Diseases (ICD‐10) procedural codes. Exclusion criteria included age younger than 18 years or older than 90 years, and diagnosis of another malignancy within 1 year before or after the diagnosis of localised RCC.

Of these patients, only patients who had been prescribed at least one antidiabetic medication prior to partial nephrectomy were included using Anatomical Therapeutic Chemical (ATC) Classification and RxNorm code. These patients were stratified into two groups based on prescription to GLP‐1RAs: those prescribed GLP‐1RAs (GLP‐1RAs group) and those who had never been prescribed GLP‐1RAs (non‐GLP‐1RAs group). The GLP‐1RAs group was 1:1 propensity score matched to the non‐GLP‐1RAs group based on baseline demographic characteristics, comorbidities, metformin and insulin prescription status, and laboratory values to adjust for potential confounding variables. Propensity score match was performed using a greedy nearest‐neighbour matching algorithm without replacement with a calliper of 0.10 SD to account for confounding variables. Variables with *p*‐values less than 0.05 were controlled for in the final propensity score match. The detailed CPT, ICD‐10, ATC and RxNorm codes are shown in Table S1.

### 2.2 Postoperative outcome variables

The primary outcomes within 90 days of PN included various complications: acute kidney injury, chronic kidney disease, pulmonary embolism, stroke, pneumonia, urinary tract infection, sepsis, myocardial infarction, arrhythmia, cardiac arrest, cardiomyopathy, transfusion, wound dehiscence, deep vein thrombosis, surgical site infection, readmission, ileus, small bowel obstruction and pneumothorax. These outcome variables were identified using the ICD‐10 codes and CPT codes. The detailed CPT and ICD‐10 codes are shown in Table S1.

### 2.3 Statistical analyses

Univariate analysis was performed to assess the influence of baseline demographic characteristics, comorbidities and laboratory values on postoperative outcomes. Baseline demographic characteristics included age at time of PN, gender, race and ethnicity. Comorbidities included essential hypertension, hyperlipidaemia, chronic ischaemic heart disease, chronic kidney disease, nicotine dependence, tobacco use and personal history of nicotine dependence. Laboratory values included body mass index (BMI), haemoglobin A1C, creatinine, urea nitrogen, eGFR, microalbuminuria and proteinuria. Metformin and insulin prescription were also included.

Differences in baseline demographic characteristics, comorbidities, metformin and insulin prescription, and laboratory values of unmatched and matched cohorts were determined using the Student *t*‐test and chi‐square test, where appropriate. A 1:1 propensity score matching was performed for the GLP‐1RAs group versus the non‐GLP‐1RAs group using the balance cohort function in TriNetX. Descriptive statistics were used to report baseline demographic characteristics, comorbidities, metformin and insulin prescription, and laboratory values, including mean, count, proportion and standard deviation.

Differences in postoperative outcomes of matched cohorts were assessed using the Student *t*‐test and risk difference, risk ratio, odds ratio (OR) and 95% confidence intervals (CI) were calculated. Overall survival was estimated using the Kaplan–Meier (KM) method and compared between matched cohorts via the log‐rank test. A multivariable Cox Proportional Hazards (CPH) model adjusted for confounding variables that may relate to overall survival, including age at PN, sex, race and comorbidities, was performed to estimate the influence of GLP‐1RAs use on overall survival. A sensitivity analysis was performed by restricting the cohort to patients from 2020 to 2025 to determine whether temporal trends influenced 90‐day postoperative outcomes. Statistical significance was defined as a *p*‐value < 0.05. All statistical analyses were performed within TriNetX. Figures were created using Microsoft Word (Microsoft Corporation), TriNetX or MATLAB.

## 3 RESULTS

A total of 44 467 patients diagnosed with localised RCC amenable to PN between 2005 and 2025 were identified in the TriNetX database. Of these patients, 10 602 (23.8%) were diagnosed with T2DM. Among the 10 602 patients with T2DM and localised RCC, 6050 were identified as having been prescribed an antidiabetic medication prior to PN: the GLP‐1RA group (*n* = 1221) and the non‐GLP‐1RAs group (*n* = 5698). Prior to matching, significant differences were observed in age at time of PN, gender (male, female, unknown), race (White, Asian), comorbidities (hypertension, hyperlipidaemia, chronic ischemic heart disease, chronic kidney disease, nicotine dependence, tobacco use, personal history of nicotine use), laboratory values (BMI, haemoglobin A1C), and metformin and insulin prescription. After matching, each cohort included 1210 patients, with no differences in baseline population characteristics except BMI and unknown gender (Table 1).

**TABLE 1:** Baseline study population characteristics of patients with type 2 diabetes mellitus undergoing partial nephrectomy before and after propensity‐score matching.

		Unmatched			1:1 Matched		
		GLP‐1RAs	Non GLP‐1RAs	*p*‐value	GLP‐1RAs	Non GLP‐1RAs	*p*‐value
*N*		1221	5698	‐	1210	1210	‐
Age at time of PN							
	Mean ± SD	61.0 ± 10.0	62.9 ± 10.6	<0.001	61.0 ± 10.0	60.6 ± 10.9	0.287
Gender							
	Male	679 (55.6%)	3661 (64.3%)	<0.001	677 (56.0%)	685 (56.6%)	0.743
	Female	541 (44.3%)	2037 (35.7%)	<0.001	532 (44.0%)	525 (43.4%)	0.774
	Unknown gender	10 (0.8%)	0 (0%)	<0.001	10 (0.8%)	0 (0%)	0.0015
Race							
	White	864 (70.8%)	3759 (66.0%)	0.001	856 (70.7%)	876 (72.4%)	0.367
	Black or African American	164 (13.4%)	767 (13.5%)	0.978	162 (13.4%)	167 (13.8%)	0.767
	Asian	42 (3.4%)	415 (7.3%)	<0.001	42 (3.5%)	34 (2.8%)	0.351
	American Indian or Alaska Native	11 (0.9%)	38 (0.7%)	0.376	11 (0.9%)	10 (0.8%)	0.827
	Native Hawaiian or Other Pacific Islander	10 (0.8%)	34 (0.6%)	0.375	10 (0.8%)	10 (0.8%)	1
	Other race	83(6.8%)	335 (5.9%)	0.222	82 (6.8%)	65 (5.4%)	0.148
	Unknown race	54 (4.4%)	350 (6.1%)	0.02	54 (4.5%)	50 (4.1%)	0.688
Ethnicity							
	Hispanic or Latino	143 (11.7%)	682 (12.0%)	0.801	141 (11.7%)	140 (11.6%)	0.949
	Not Hispanic or Latino	937 (76.7%)	4280 (75.1%)	0.231	929 (76.8%)	919 (76.0%)	0.632
	Unknown ethnicity	141 (11.5%)	736 (12.9%)	0.192	140 (11.6%)	151 (12.5%)	0.492
Comorbidity							
	Essential hypertension	974 (79.8%)	3880 (68.1%)	<0.001	963 (79.6%)	963 (79.6%)	1
	hyperlipidaemia	724 (59.3%)	2427 (42.6%)	<0.001	713 (58.9%)	708 (58.5%)	0.836
	Chronic ischemic heart disease	361 (29.6%)	1249 (21.9%)	<0.001	354 (29.3%)	336 (27.8%)	0.418
	Chronic kidney disease	305 (25.0%)	1102 (19.3%)	<0.001	300 (24.8%)	300 (24.8%)	1
	Nicotine dependence	199 (16.3%)	789 (13.8%)	0.026	198 (16.4%)	180 (14.9%)	0.314
	Tobacco use	70 (5.7%)	227 (4.0%)	0.006	69 (5.7%)	61 (5.0%)	0.471
	Personal history of nicotine dependence	289 (23.7%)	956 (16.8%)	<0.001	281 (23.2%)	265 (21.9%)	0.436
Lab values							
BMI	*n* (%)	970 (79.4%)	4413 (77.4%)		959 (79.3%)	973 (80.4%)	
	Mean ± SD	34.3 ± 6.7	32.3 ± 6.8	<0.001	34.3 ± 6.8	33.2 ± 7.2	0.001
Haemoglobin A1c/haemoglobin. total in blood	*n* (%)	769 (63.0%)	2641 (46.3%)		758 (62.6%)	760 (62.8%)	
	Mean ± SD	7.3 ± 1.7	7.1 ± 1.7	0.033	7.3 ± 1.7	7.3 ± 1.8	0.927
Creatinine (mass/volume) in serum, plasma or blood	*n* (%)	996 (81.6%)	4712 (82.7%)		985 (81.4%)	1077 (89.0%)	
	Mean ± SD	1.1 ± 0.7	1.2 ± 3.6	0.188	1.1 ± 0.7	1.3 ± 5.2	0.255
Urea nitrogen (mass/volume) in serum, plasma or blood	*n* (%)	984 (80.6%)	4575 (80.3%)		973 (80.4%)	1051 (86.9%)	
	Mean ± SD	19.6 ± 10.0	19.7 ± 10.2	0.78	19.6 ± 10.0	18.9 ± 9.5	0.102
Glomerular filtration rate/1.73 m^2^ predicted (volume rate/area) in serum, plasma or blood by creatinine‐based formula (MDRD)	*n* (%)	996 (81.6%)	4712 (82.7%)		985 (81.4%)	1.074 (88.8%)	
	Mean ± SD	74.2 ± 27.9	73.0 ± 28.2	0.22	74.2 ± 27.9	74.1 ± 28.6	0.947
Microalbumin (mass/volume) in urine	*n* (%)	408 (33.4%)	783 (13.7%)		401 (33.1%)	250 (20.7%)	
	Mean ± SD	32.7 ± 196.0	34.9 ± 172.7	0.847	33.2 ± 197.6	30.8 ± 95.3	0.86
Protein (mass/volume) in urine	*n* (%)	270 (22.1%)	877 (15.4%)		268 (22.1%)	247 (20.4%)	
	Mean ± SD	45.8 ± 38.5	43.6 ± 37.5	0.386	45.7 ± 38.5	44.9 ± 37.6	0.813
Medication							
	Metformin	898 (73.5%)	2996 (52.6%)	<0.001	887 (73.3%)	877 (72.5%)	0.647
	Insulin	706 (57.8%)	2171 (38.1%)	<0.001	695 (57.4%)	682 (56.4%)	0.594

The 90‐day postoperative adverse outcomes were compared between the GLP‐1RAs group and the non‐GLP‐1RAs group. The patients in the GLP‐1RAs group had a reduced risk of the following 90‐day postoperative adverse outcomes: acute kidney injury (risk difference [RD] = −0.028, risk ratio [RR] = 0.734, odds ratio [OR] = 0.712), arrhythmia (RD = −0.026, RR = 0.746, OR = 0.725), readmission (RD = −0.045, RR = 0.852, OR = 0.8) and ileus (RD = −0.019, RR = 0.343, OR = 0.336) compared to patients in the non‐GLP‐1RAs group (*p* < 0.05 for each) (Table 2).

**TABLE 2:** Ninety‐day post‐partial nephrectomy adverse outcomes.

1:1 Matched						
Outcomes	GLP‐1RAs (*n* = 1210)	Non GLP‐1RAs (*n* = 1210)	Risk difference (95% CI)	*p*‐value	Risk ratio (95% Cl)	Odds ratio (95% CI)
Acute kidney injury	94 (7.8%)	128 (10.6%)	−0.028 (−0.051, −0.005)	0.017	0.734 (0.57, 0.947)	0.712 (0.539, 0.941)
Chronic kidney disease	214 (17.7%)	213 (17.6%)	0.001 (−0.03, 0.031)	0.957	1.005 (0.846, 1.193)	1.006 (0.816, 1.24)
Pulmonary embolism	13 (1.1%)	15 (1.2%)	−0.002 (−0.01, 0.007)	0.704	0.867 (0.414, 1.814)	0.865 (0.41, 1.826)
Stroke	20 (1.7%)	19 (1.6%)	0.001 (−0.009, 0.011)	0.872	1.053 (0.565, 1.962)	1.054 (0.559, 1.984)
Pneumonia	20 (1.7%)	23 (1.9%)	−0.002 (−0.013, 0.008)	0.644	0.870 (0.48, 1.575)	0.867 (0.474, 1.588)
Urinary tract infection	71 (5.9%)	67 (5.5%)	0.003 (−0.015, 0.022)	0.726	1.06 (0.766, 1.465)	1.063 (0.754, 1.5)
Sepsis	25 (2.1%)	29 (2.4%)	−0.003 (−0.015, 0.008)	0.582	0.862 (0.508, 1.463)	0.859 (0.5, 1.476)
Myocardial infarction	16 (1.3%)	19 (1.6%)	−0.002 (−0.012, 0.007)	0.609	0.842 (0.435, 1.63)	0.84 (0.43, 1.641)
Arrhythmia	91 (7.5%)	122 (10.1%)	−0.026 (−0.048, −0.003)	0.026	0.746 (0.575, 0.967)	0.725 (0.546, 0.963)
Cardiac arrest	‐	‐	‐	‐	‐	‐
Cardiomyopathy	17 (1.4%)	26 (2.1%)	−0.007 (−0.018, 0.003)	0.166	0.654 (0.357, 1.199)	0.649 (0.35, 1.202)
Transfusion	21 (1.7%)	33 (2.7%)	−0.01 (−0.022, 0.002)	0.099	0.636 (0.37, 1.093)	0.63 (0.362, 1.095)
Wound dehiscence	13 (1.1%)	12 (1.0%)	0.001 (−0.007, 0.009)	0.841	1.083 (0.496, 2.365)	1.084 (0.493, 2.386)
Deep vein thrombosis	17 (1.4%)	20 (1.7%)	−0.002 (−0.012, 0.007)	0.619	0.85 (0.447,1.615)	0.848 (0.442, 1.627)
Surgical site infection	18 (1.5%)	24 (2.0%)	−0.559 (−1.6,0.481)	0.2923	0.731(0.407,1.313)	0.727 (0.4,1.32)
Readmission	317 (26.2%)	372 (30.7%)	−0.045 (−0.081, −0.01)	0.013	0.852 (0.751, 0.967)	0.8 (0.67, 0.955)
Ileus	12 (1.0%)	35 (2.9%)	−0.019 (−0.03, −0.008)	0.001	0.343 (0.179, 0.657)	0.336 (0.174, 0.651)
Small bowel obstruction	‐	‐	‐	‐	‐	‐
Pneumothorax	‐	‐	‐	‐	‐	‐

In the sensitivity analysis restricted to 2020–2025, the GLP‐1RA group continued to demonstrate a decreased risk of acute kidney injury (RD = −0.038, RR = 0.638, OR = 0.612, *p* = 0.014) and readmission (RD = −0.05, RR = 0.837, OR = 0.781, *p* = 0.044) compared with the non‐GLP‐1RA group. Although not statistically significant, the GLP‐1RA group also demonstrated a trend towards a decreased risk of arrhythmias (RD = −0.02, RR = 0.783, OR = 0.767). The analysis for ileus was limited by an insufficient number of events to generate results (Table S2).

In the 1:1 propensity score matched cohort, the median follow‐up was 665 days for the GLP‐1RAs group and 1161 days for the non GLP‐1RAs group. Kaplan–Meier survival analyses and log‐rank tests were conducted over 2‐ and 3‐year time frames. At 2 years, the GLP‐1RAs group had 97.28% survival probability, compared to 94.29% in the non‐GLP‐1RAs group, corresponding to significantly improved survival (*χ* ^2^ = 9.57, *df* = 1, *p* = 0.002) (Figure 2). At 3 years, survival probabilities were 96.10% and 91.96% for the GLP‐1RAs and non‐GLP‐1RAs, respectively, reaching a significant difference (*χ* ^2^ = 11.918, *df* = 1, *p* = 0.001) (Figure 3). TriNetX automatically censors patients once they no longer contribute relevant information to the analysis.

Overall survival was estimated using a multivariable CPH model (Table 3). Increased age at the time of PN was associated with higher mortality (HR = 1.037, *p* < 0.0001). Race and sex did not significantly influence survival. Comorbidities such as chronic ischaemic heart disease (HR = 1.46, *p* < 0.0001), chronic kidney disease (HR = 1.839, *p* < 0.0001) and nicotine dependence (HR = 1.487, *p* = 0.0009) were all associated with worse overall survival compared with those without these diagnoses. Use of GLP‐1RAs was associated with significantly improved overall survival (HR = 0.665, *p* = 0.01).

**TABLE 3:** Cox proportional hazard model for overall survival.

Variables	Hazard ratio (95% CI)	*p*‐value
GLP‐1RAs (ref = non‐GLP‐1RAs)	0.665 (0.488, 0.907)	0.01
Age at partial nephrectomy	1.037 (1.027, 1.046)	< 0.0001
Male (ref = Female)	1.086 (0.915, 1.289)	0.345
American Indian or Alaska native	1.455 (0.497, 4.257)	0.494
Asian	0.92 (0.524, 1.616)	0.773
Black or African American	0.997 (0.618, 1.610)	0.992
Native Hawaiian or other Pacific Islander	1.449 (0.614, 3.417)	0.504
Other race	0.779 (0.375, 1.620)	0.504
White	1.28 (0.824, 1.989)	0.272
Essential hypertension	1.113 (0.899, 1.378)	0.326
Chronic Ischaemic heart disease	1.46 (1.216, 1.754)	< 0.0001
Hyperlipidaemia	1.008 (0.837, 1.213)	0.936
Chronic kidney disease	1.839 (1.535, 2.204)	< 0.0001
Nicotine dependence	1.487 (1.178, 1.879)	0.0009
Tobacco use	1.171 (0.807, 1.701)	0.406
Personal history of nicotine dependence	1.196 (0.973, 1.469)	0.089

## 4 DISCUSSION

**FIGURE 1:**
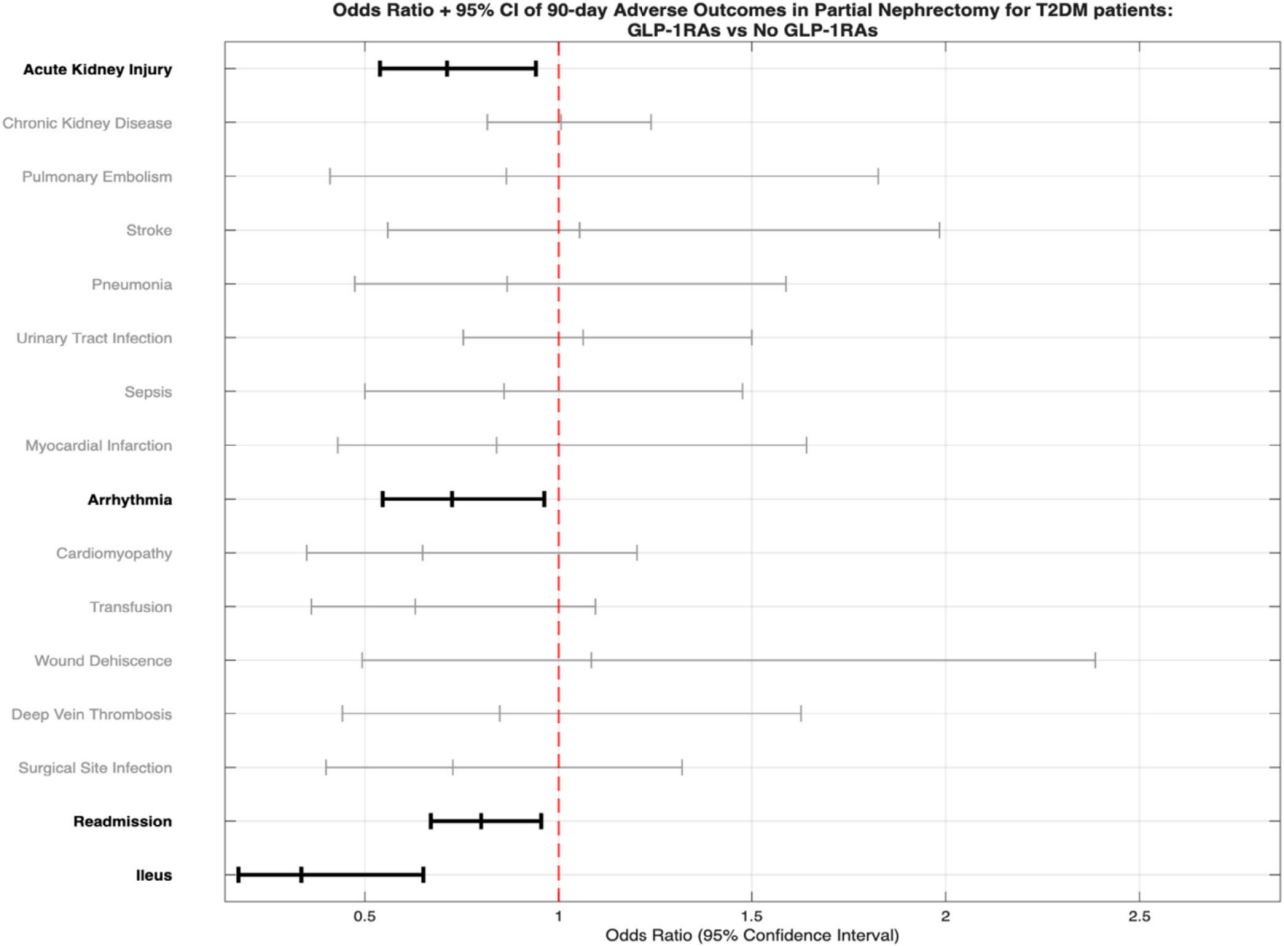
Forest plot of odds ratios (OR) and 95% confidence intervals (CI) for post–partial nephrectomy adverse outcomes in GLP‐1RAs and non‐GLP‐1RAs groups.

**FIGURE 2:**
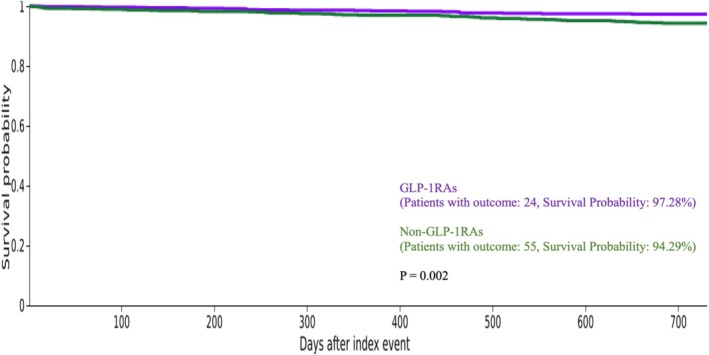
Kaplan–Meier plot of 2‐year overall survival post partial nephrectomy.

**FIGURE 3:**
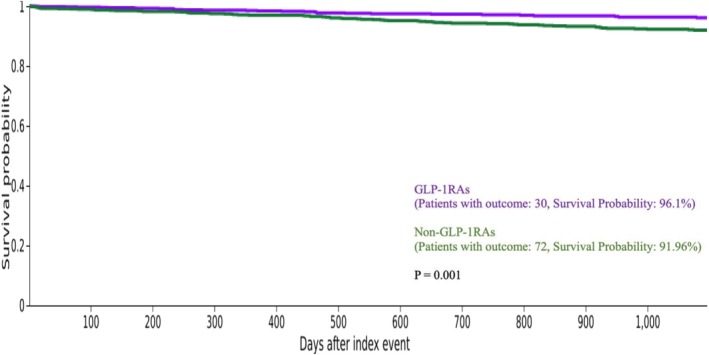
Kaplan–Meier plot of 3‐year overall survival post partial nephrectomy.

In this large, propensity score‐matched analysis of patients with T2DM undergoing PN, we found that perioperative use of GLP‐1RA was linked to significantly better clinical outcomes. After adjusting for significant baseline differences in demographics and comorbidities, patients taking GLP‐1RAs experienced fewer 90‐day postoperative adverse events (Figure 1) and showed improved 2‐ and 3‐year overall survival (Figures 2 and 3, respectively). These findings emphasise a strong protective effect of this medication class in a high‐risk surgical population.

### 4.1 Acute kidney injury

To explore the factors of these improved outcomes, we examined 90‐days postoperative complications. Our results indicated that GLP‐1RA use was associated with significantly lower incidence of acute kidney injury (AKI), reflecting a 2.8% absolute risk reduction in AKI among the GLP‐1RAs group (7.8%) compared to the non‐GLP‐1RAs group (10.6%). This finding aligns with a growing body of literature supporting the renoprotective effects of GLP‐1RAs. These renoprotective effects of GLP‐1RAs are likely exerted through haemodynamic and hormonal pathways. These agents inhibit the sodium‐hydrogen exchanger 3 (NHE3) in the proximal tubule, promoting natriuresis and lowering blood pressure.^11^ They also suppress the renin‐angiotensin‐aldosterone system (RAAS), improving volume status and reducing intraglomerular pressure.^11^ Additionally, GLP‐1RAs reduce oxidative stress and inflammation through downregulation of NF‐κB signalling and suppression of pro‐inflammatory cytokines.^12^ Collectively, these mechanisms may mitigate renal ischaemia and oxidative stress during surgery. Large randomised clinical trials have provided further evidence of the renoprotective effects of GLP‐1RAs. The FLOW trial, which included 3533 participants with T2DM and chronic kidney disease (CKD), revealed a slower annual decline in eGFR among patients receiving semaglutide compared to control.^10^ Similarly, the SELECT trial, which enrolled over 17 500 participants with obesity and CVD (without diabetes), reported a significantly slower rate of eGFR decline in the semaglutide arm.^13^ Together with our results, these findings suggest that GLP‐1RAs may confer protection against AKI in the perioperative setting of partial nephrectomy for RCC.

### 4.2 Arrhythmia

We observed decreased odds of postoperative arrhythmias among patients receiving GLP‐1RAs after PN with an incidence of 7.5% in the GLP‐1RAs group compared with 10.1% in the non‐GLP‐1RAs group. Although no previous studies have specifically examined this surgical subgroup, our findings suggest a potential cardioprotective effect. Postoperative arrhythmias are a known complication in patients with T2DM; however, mounting evidence indicates that GLP‐1RAs do not contribute to this risk.^14^, ^15^ Large meta‐analyses and observational studies in T2DM populations, including patients with CKD and those in perioperative settings, have shown similar results.^14^, ^15^, ^16^, ^17^ Moreover, GLP‐1RAs have been associated with improved surgical outcomes—such as lower 30‐day readmission rates, fewer wound complications, and reduced incidence of pneumonia—without evidence of increased cardiac arrhythmias.^16^, ^18^ Their overall cardiovascular and renal benefits, including improved glycaemic control, weight loss, blood pressure reduction and anti‐inflammatory effects, likely contribute to the lower arrhythmia risk observed.^16^ Taken together, our study adds to the growing evidence of the cardioprotective effects of GLP‐1RAs and underscores their potential to reduce postoperative arrhythmias in T2DM patients undergoing PN.

### 4.3 Readmission

GLP‐1RA use was associated with a significantly lower 90‐day readmission rate after PN for localised RCC with rates of 26.2% in the GLP‐1RA group versus 30.7% in the non‐GLP‐1RA group, reflecting a 4.5% absolute risk reduction. Readmission after PN is generally uncommon, with prior studies reporting 30‐day rates of 4.2–5.9%.^19^, ^20^ While our findings indicate a higher rate of readmission in this cohort compared with prior reports, diabetes is an independent risk factor for readmission, with a National Surgical Quality Improvement Program Database (NSQIP) study showing higher odds of readmission in diabetic versus non‐diabetic patients (OR 1.6), largely driven by complications such as surgical site infection, renal failure, UTI, transfusion, DVT and sepsis.^20^ Surgical approach also influences risk, with robotic/laparoscopic PN outperforming open surgery. Evidence from broader surgical cohorts further suggests that GLP‐1RA use is associated with lower 30‐day readmission rates across procedure types, including urologic surgeries.^16^ Our study extends these findings by demonstrating a reduced 90‐day readmission risk in diabetic patients specifically undergoing PN, supporting a potential protective role of GLP‐1RAs against postoperative readmission.

### 4.4 Ileus

Our results indicated that GLP‐1RA use was associated with lower odds of postoperative ileus, occurring in 1.0% of the GLP‐1RA cohort compared with 2.9% of the non‐GLP‐1RA cohort, reflecting a 1.9% absolute reduction. Initially, this finding appears paradoxical because GLP‐1RAs are known to slow intestinal transit and impair gastric emptying.^21^ However, in patients with T2DM, impaired gastric emptying is also linked to hyperglycaemia^22^ and diabetic neuropathy, disrupting vagus nerve function in the gut.^23^ Studies have shown that GLP‐1RAs are known to improve both glycaemic control^9^ and diabetic neuropathy,^24^, ^25^ which may mitigate vagal dysfunction contributing to ileus. Therefore, the observed reduction in ileus risk may reflect GLP‐1RAs' metabolic and autonomic benefits rather than a direct gut promotility effect. Moreover, GLP‐1RAs are often withheld 1 week prior to surgery per multi‐society clinical practice guideline,^26^ which further supports the idea that the protective association is more likely because of enhanced preoperative glycaemic control and autonomic function rather than pharmacological impact on gastric motility.

Importantly, a sensitivity analysis limited to patients treated between 2020 and 2025 showed significantly lower rates of acute kidney injury and readmission, as well as a trend towards reduced arrhythmias in the GLP‐1RA group. This may suggest that these associations are unlikely to be solely explained by improvements in a contemporary surgical era, such as an increased use of robotic minimally invasive surgery or enhanced perioperative care.

### 4.5 Overall survival

Kaplan–Meier analysis demonstrated significantly improved overall survival among GLP‐1RA users compared to non‐users, despite shorter median follow‐up (665 vs 1161 days). Survival rates remained higher at both 2 years (97.28% vs 94.29%) and 3 years (96.1% vs 91.96%). A multivariable CPH model controlling for age at the time of PN, sex, race and comorbidities demonstrated that the use of GLP‐1RAs was associated with significantly improved overall survival (HR = 0.665, *p* = 0.01). To our knowledge, this is the first study to evaluate GLP‐1RA use and survival in T2DM patients with localised RCC after PN. These findings align with emerging evidence that GLP‐1RAs exert systemic benefits beyond glycaemic control. Propensity matching minimised confounders, including metformin use, which is independently associated with improved survival.^6^, ^7^, ^27^ Experimental work further supports a role for glucagon‐like peptide‐1 receptor (GLP‐1R) in RCC biology, with studies reporting survival benefits in clear cell but not papillary subtypes.^28^ The variability in outcomes across different cancers underscores the complex, tumour microenvironment‐dependent nature of GLP‐1R effects on tumour biology. Taken together, our study contributes to the growing recognition of an association between GLP‐1RA use and improved overall survival in cancer populations.

## 5 LIMITATIONS

The results reported in this study should be interpreted with caution. The study was limited by the use of a retrospective database that relies on the accuracy of coding and reporting. Because retrospective databases are susceptible to reporting bias, the observed complication rates and the estimated risk ratio may be affected. We aimed to specifically investigate clinical T1–T2 RCC by excluding patients coded as T3+, N1+ or M1. However, because of incomplete reporting of TNM staging in TriNetX, only a small proportion of the 332 568 patients with renal cell carcinoma had staging information available: 9451 (2.8%) for T stage, 9016 (2.7%) for N stage and 7741 (2.3%) for M stage. Consequently, some patients with more advanced disease may still be included. We attempted to mitigate this limitation by focussing on patients treated with partial nephrectomy, a procedure typically reserved for localised RCC. Although we observed a trend in postoperative adverse events and overall survival, we were unable to assess medication adherence or duration. Moreover, socioeconomic status, ischaemia time, tumour size, hospital type, and surgical modality (i.e. open, laparoscopic, robotic)—potential unmeasured confounding variables that may influence postoperative outcomes and overall survival—were not available and thus could not be accounted for. Lastly, T2DM has been linked to hypogonadism, particularly in male patients. A recent study demonstrated that hypogonadism in men is associated with frailty, which has been linked to adverse postoperative outcomes following PN.^29^ Because our study includes both male and female patients, we were unable to account for endocrine dysfunction. Therefore, endocrine dysfunction may represent an additional source of unmeasured confounding variable. Future studies should consider controlling for potential unmeasured confounding variables in analysing postoperative outcomes and overall survival.

The rapid and substantial rise in GLP‐1RA utilisation is poised to significantly impact the medical care landscape across multiple medical specialties. This study is the first to leverage a large‐scale database to study postoperative adverse events and overall survival in patients with DM taking GLP‐1RA who underwent PN. Use of GLP‐1RA was found to be associated with reduced risk of certain 90‐day post PN medical complications. The findings of this study have significant implications for perioperative management, risk stratification and long‐term outcomes in this patient population.

## CONFLICT OF INTEREST STATEMENT

All authors declared that there are no conflicts of interest.

## Supporting information


**Table S1.** Code and Code Descriptions.
Table S2*. Ninety‐Day Post‐Partial Nephrectomy Adverse Outcomes (2020–2025)*.

## Data Availability

The data used in this study were sourced from TriNetX, a third‐party research database, which are not publicly accessible and thus cannot be provided. However, the patient selection process and study replication are possible by adhering to the methodology described in this study.
