# Supplementary material for: GLP‐1RA use improves outcomes post partial nephrectomy in T2DM patients with RCC: A TriNetX study
Source: BJUI Compass. 2026 Feb 2;7(2):e70169. doi: 10.1002/bco2.70169 (PMC12863992; doi:10.1002/bco2.70169)
Supplement: Supplementary file 1 — Table S1. Code and Code Descriptions. Table S2. Ninety‐Day Post‐Partial Nephrectomy Adverse Outcomes (2020–2025). [file BCO2-7-e70169-s001.docx]

**Supplemental Table 1. Code and Code Descriptions**

| **Variable** | **Code** | **Code Description** |
| --- | --- | --- |
| Type 2 Diabetes Mellitus | UMLS:ICD10CM:E11 | Type 2 diabetes mellitus |
| Localized Renal Cell Carcinoma | UMLS:ICD10CM:C64 | Malignant neoplasm of kidney, except renal pelvis |
| Partial Nephrectomy | UMLS:ICD10PCS:0TB00ZZ | Excision of Right Kidney, Open Approach |
|  | UMLS:ICD10PCS:0TB10ZZ | Excision of Left Kidney, Open Approach |
|  | UMLS:ICD10PCS:0TB04ZZ | Excision of Right Kidney, Percutaneous Endoscopic Approach |
|  | UMLS:ICD10PCS:0TB14ZZ | Excision of Left Kidney, Percutaneous Endoscopic Approach |
|  | UMLS:ICD10PCS:0TB03ZZ | Excision of Right Kidney, Percutaneous Approach |
|  | UMLS:ICD10PCS:0TB13ZZ | Excision of Left Kidney, Percutaneous Approach |
|  | UMLS:ICD10PCS:0TB07ZZ | Excision of Right Kidney, Via Natural or Artificial Opening |
|  | UMLS:ICD10PCS:0TB17ZZ | Excision of Left Kidney, Via Natural or Artificial Opening |
|  | UMLS:ICD10PCS:0TB08ZZ | Excision of Right Kidney, Via Natural or Artificial Opening Endoscopic |
|  | UMLS:ICD10PCS:0TB18ZZ | Excision of Left Kidney, Via Natural or Artificial Opening Endoscopic |
|  | UMLS:CPT:50240 | Nephrectomy, partial |
|  | UMLS:CPT:50543 | Laparoscopy, surgical; partial nephrectomy |
| Medication | NLM:ATC:A10BJ | Glucagon-like peptide-1 (GLP-1) analogues |
|  | NLM:ATC:A10A | INSULINS AND ANALOGUES |
|  | NLM:ATC:A10BA | Biguanides |
|  | NLM:ATC:A10BB | Sulfonylureas |
|  | NLM:ATC:A10BF | Alpha glucosidase inhibitors |
|  | NLM:ATC:A10BG | Thiazolidinediones |
|  | NLM:ATC:A10BH | Dipeptidyl peptidase 4 (DPP-4) inhibitors |
|  | NLM:ATC:A10BK | Sodium-glucose co-transporter 2 (SGLT2) inhibitors |
|  | NLM:ATC:A10BX | Other blood glucose lowering drugs, excl. insulins |
|  | NLM:RXNORM:6809 | metformin |
|  | NLM:ATC:C10AC | Bile acid sequestrants |
|  | NLM:RXNORM:1760 | bromocriptine |
|  | NLM:RXNORM:274332 | nateglinide |
|  | NLM:RXNORM:73044 | repaglinide |
| Acute Kidney Injury | UMLS:ICD10CM:N17 | Acute kidney failure |
| Chronic Kidney Disease | UMLS:ICD10CM:N18.6 | End stage renal disease |
|  | UMLS:ICD10CM:N18.9 | Chronic kidney disease, unspecified |
|  | UMLS:ICD10CM:N18.3 | Chronic kidney disease, stage 3 (moderate) |
|  | UMLS:ICD10CM:N18.5 | Chronic kidney disease, stage 5 |
|  | UMLS:ICD10CM:N18.4 | Chronic kidney disease, stage 4 (severe) |
|  | UMLS:ICD10CM:N18.1 | Chronic kidney disease, stage 1 |
|  | UMLS:ICD10CM:N18.2 | Chronic kidney disease, stage 2 (mild) |
| Pulmonary Embolism | UMLS:ICD10CM:I26 | Pulmonary embolism |
| Stroke | UMLS:ICD10CM:I63 | Cerebral infarction |
|  | UMLS:ICD10CM:I62 | Other and unspecified nontraumatic intracranial hemorrhage |
| Pneumonia | UMLS:ICD10CM:J12 | Viral pneumonia, not elsewhere classified |
|  | UMLS:ICD10CM:J13 | Pneumonia due to Streptococcus pneumoniae |
|  | UMLS:ICD10CM:J14 | Pneumonia due to Hemophilus influenzae |
|  | UMLS:ICD10CM:J15 | Bacterial pneumonia, not elsewhere classified |
|  | UMLS:ICD10CM:J16 | Pneumonia due to other infectious organisms, not elsewhere classified |
|  | UMLS:ICD10CM:J17 | Pneumonia in diseases classified elsewhere |
|  | UMLS:ICD10CM:J18 | Pneumonia, unspecified organism |
| Urinary Tract Infection | UMLS:ICD10CM:N39.0 | Urinary tract infection, site not specified |
|  | UMLS:ICD10CM:N10 | Acute pyelonephritis |
|  | UMLS:ICD10CM:N30.0 | Acute cystitis |
|  | UMLS:ICD10CM:N30.9 | Cystitis, unspecified |
| Sepsis | UMLS:ICD10CM:A41 | Other sepsis |
|  | UMLS:ICD10CM:R65.2 | Severe sepsis |
|  | UMLS:ICD10CM:T81.44 | Sepsis following a procedure |
| Myocardial Infarction | UMLS:ICD10CM:I21 | Acute myocardial infarction |
|  | UMLS:ICD10CM:I22 | Subsequent ST elevation (STEMI) and non-ST elevation (NSTEMI) myocardial infarction |
| Arrythmias | UMLS:ICD10CM:I48 | Atrial fibrillation and flutter |
|  | UMLS:ICD10CM:I49 | Other cardiac arrhythmias |
| Cardiac Arrest | UMLS:ICD10CM:I46 | Cardiac arrest |
| Cardiomyopathy | UMLS:ICD10CM:I42 | Cardiomyopathy |
| Transfusion | UMLS:ICD10PCS:302 | Transfusion |
| Wound Dehiscence | UMLS:ICD10CM:T81.30 | Disruption of wound, unspecified |
|  | UMLS:ICD10CM:T81.32 | Disruption of internal operation (surgical) wound, not elsewhere classified |
|  | UMLS:ICD10CM:T81.31 | Disruption of external operation (surgical) wound, not elsewhere classified |
| Deep Vein Thrombosis | UMLS:ICD10CM:I82.4 | Acute embolism and thrombosis of deep veins of lower extremity |
| Surgical Site Infection | UMLS:ICD10CM:T81.40 | Infection following a procedure, unspecified |
|  | UMLS:ICD10CM:T81.41 | Infection following a procedure, superficial incisional surgical site |
|  | UMLS:ICD10CM:T81.42 | Infection following a procedure, deep incisional surgical site |
|  | UMLS:ICD10CM:T81.43 | Infection following a procedure, organ and space surgical site |
|  | UMLS:ICD10CM:T81.49 | Infection following a procedure, other surgical site |
| Readmission | UMLS:CPT:1013659 | Hospital Inpatient and Observation Care Services |
|  | UMLS:HL7V3.0:VisitType:ACUTE | Visit: Inpatient Acute |
|  | UMLS:HL7V3.0:VisitType:IMP | Visit: Inpatient Encounter |
|  | UMLS:HL7V3.0:VisitType:NONAC | Visit: Inpatient Non-acute |
| Ileus | UMLS:ICD10CM:K56.7 | Ileus, unspecified |
|  | UMLS:ICD10CM:K56.0 | Paralytic ileus |
| Small Bowel Obstruction | UMLS:ICD10CM:K56.6 | Other and unspecified intestinal obstruction |
| Pneumothorax | UMLS:ICD10CM:J93 | Pneumothorax and air leak |

**Supplemental Table *2. Ninety-Day Post-Partial Nephrectomy Adverse Outcomes (2020-2025)***

| **1:1 Matched** | | | | | | |
| --- | --- | --- | --- | --- | --- | --- |
| **Outcomes** | **GLP-1RAs**  **(n = 663)** | **Non GLP-1RAs**  **(n = 663)** | **Risk Difference**  **(95% CI)** | **P-value** | **Risk Ratio**  **(95% Cl)** | **Odds Ratio (95% CI)** |
| **Acute Kidney Injury** | **44 (6.6 %)** | **69 (10.4%)** | **-0.038**  **(-0.068, -0.008)** | **0.014** | **0.638**  **(0.444, 0.916)** | **0.612 (0.412, 0.908)** |
| Chronic Kidney Disease | 126 (19.0%) | 128 (19.3%) | -0.003  (-0.045, 0.039) | 0.889 | 0.984  (0.789, 1.228) | 0.981 (0.746, 1.289) |
| Pulmonary Embolism | - | - | - | - | - | - |
| Stroke | - | - | - | - | - | - |
| Pneumonia | - | - | - | - | - | - |
| Urinary Tract Infection | 31 (4.7%) | 36 (5.4%) | -0.008  (-0.031, 0.016) | 0.531 | 0.861  (0.539, 1.375) | 0.854 (0.522, 1.398) |
| Sepsis | 11 (1.7%) | 19 (2.9%) | -0.012  (-0.028, 0.004) | 0.14 | 0.579   (0.278, 1.207) | 0.572 (0.27, 1.211) |
| Myocardial Infarction | - | - | - | - | - | - |
| Arrhythmia | 47 (7.1%) | 60 (0.09%) | -0.02   (-0.049, -0.01) | 0.19 | 0.783   (0.543, 1.13) | 0.767 (0.515, 1.142) |
| Cardiac Arrest | - | - | - | - | - | - |
| Cardiomyopathy | - | - | - | - | - | - |
| Transfusion | - | - | - | - | - | - |
| Wound Dehiscence | - | - | - | - | - | - |
| Deep Vein Thrombosis | - | - | - | - | - | - |
| Surgical Site Infection | 10 (1.5%) | 15 (2.3%) | -0.008  (-0.022,0.007) | 0.313 | 0.667(0.302,1.473) | 0.662 (0.295,1.483) |
| **Readmission** | **170 (25.6%)** | **203 (30.6%)** | **-0.05**  **(-0.098, -0.001)** | **0.044** | **0.837**  **(0.704, 0.996)** | **0.781 (0.615, 0.993)** |
| Ileus | - | - | - | - | - | - |
| Small Bowel Obstruction | - | - | - | - | - | - |
| Pneumothorax | - | - | - | - | - | - |
